# Interpreting protein variant effects with computational predictors and deep mutational scanning

**DOI:** 10.1242/dmm.049510

**Published:** 2022-06-23

**Authors:** Benjamin J. Livesey, Joseph A. Marsh

**Affiliations:** MRC Human Genetics Unit, Institute of Genetics and Cancer, University of Edinburgh, Edinburgh EH4 2XU, UK

**Keywords:** Benchmarking, Circularity, Deep mutational scan, Machine learning, Multiplexed assay of variant effect, Variant effect predictor

## Abstract

Computational predictors of genetic variant effect have advanced rapidly in recent years. These programs provide clinical and research laboratories with a rapid and scalable method to assess the likely impacts of novel variants. However, it can be difficult to know to what extent we can trust their results. To benchmark their performance, predictors are often tested against large datasets of known pathogenic and benign variants. These benchmarking data may overlap with the data used to train some supervised predictors, which leads to data re-use or circularity, resulting in inflated performance estimates for those predictors. Furthermore, new predictors are usually found by their authors to be superior to all previous predictors, which suggests some degree of computational bias in their benchmarking. Large-scale functional assays known as deep mutational scans provide one possible solution to this problem, providing independent datasets of variant effect measurements. In this Review, we discuss some of the key advances in predictor methodology, current benchmarking strategies and how data derived from deep mutational scans can be used to overcome the issue of data circularity. We also discuss the ability of such functional assays to directly predict clinical impacts of mutations and how this might affect the future need for variant effect predictors.

## Abstract (precis)

**Summary:** Variant effect predictors (VEPs) are algorithms that aim to predict the likelihood that a genetic or protein variant will be pathogenic. Herein, we review benchmarking procedures and deep mutational scanning as methods for validating VEPs.

## Introduction

Rapid advances in sequencing technology over the past two decades has resulted in genomic information becoming an integral tool in both research and clinical fields. This wealth of data has helped identify thousands of human genetic variants in the population (Karczewski et al., 2020). A recent whole-exome sequencing study of the UK biobank cohort (Bycroft et al., 2018) identified a median of almost 20,000 coding variants per participant (Backman et al., 2021). However, most genetic variants are benign and unrelated to any disease. Variant effect predictors (VEPs) are computational tools that use this information to predict the phenotypic outcome of genetic variants and help highlight variants that are most likely to have clinically relevant effects. Where the phenotypic impact of a variant is uncertain, the variant is classified as a ‘variant of uncertain (clinical) significance’ (VUS) (Richards et al., 2015). VUSs account for a high proportion of identified variants (Balmaña et al., 2016), an issue that VEPs can potentially help to address. VEPs provide a quick, free and scalable alternative to time-consuming and expensive mutagenesis studies required to confirm the phenotypic effect of a VUS. This is particularly applicable to rare variants, where the expense of wet-lab experiments may be difficult to justify. Since the development of Sorting Intolerant From Tolerant (SIFT) (Ng and Henikoff, 2001), many other VEPs have been published with varying methodologies.

However, many of these VEPs provide contradictory results when classifying identical variants (Miller et al., 2019). This underscores the need for useful and accurate VEP benchmarking (Box 1, Glossary), which is becoming increasingly important as state-of-the-art predictors emerge. The authors of VEPs commonly assess performance against several previously published predictors using established variant databases. However, data circularity (Box 1) is an unresolved source of bias for many methods of benchmarking (Grimm et al., 2015). Comparisons between predictors often introduce bias by assessing predictor performance against the same data that were used to train them. Therefore, robust and unbiased benchmarking by independent groups is essential for assessing the performances of different VEPs.

One solution to this issue of data circularity is the use of independent variant effect datasets from deep mutational scanning (DMS; Box 1) experiments. DMS is a high-throughput technique to generate functional scores for, potentially, all variants of a protein (Fowler and Fields, 2014). For the purposes of benchmarking, DMS is fully independent from most training data. Most of the fitness scores (Box 1) from a DMS experiment are novel and not present in any sequencing dataset and, therefore, will not have been used to train or test previous predictors. Even those mutants that do exist in current datasets are scored independently of their previous categorisation.

In this Review, we will discuss the progress made in VEP methodology and assess different benchmarking strategies with an emphasis on the usage of DMS data and other large functional assays. We will also discuss the impact DMS may have as a direct independent measure of variant effects. We focus on VEPs developed to predict whether mutations are likely to be causing disease, excluding those that have been developed specifically to predict the effects of mutations on specific biophysical properties, such as protein stability (Gerasimavicius et al., 2020) or protein-protein interactions (Janin et al., 2003; Rodrigues et al., 2019). We hope that some of the issues we highlight will inform future VEP benchmarking efforts and the use of functional datasets.

**Box 1. Glossary**

**Bagging:** A model-construction approach, most commonly used in random forest algorithms (see Box 2). Bagging creates a large ensemble of models, each of which only sees a subset of training data and a subset of input features. Although each model only has a part of the full picture, taken together they can correct each other's biases.

**Benchmarking:** Evaluation by comparison against a standard.

**Conservation:** When a particular amino acid is present in the same aligned position across a high proportion of related proteins, it is highly conserved. Conservation is an important predictive feature for many VEPs. Some predictors also use conservation at the nucleotide level.

**Curated benchmarking datasets:** Although large numbers of variants are available to train predictors, best results are usually obtained from high-quality datasets of validated variants. For this reason databases, such as VariBench (http://structure.bmc.lu.se/VariBench/) (Nair and Vihinen, 2013) exist, which curate the included variants to ensure high quality.

**Data circularity:** The re-use of data used to train a predictor, in order to assess the same predictor. Grimm et al. (2015) identified two types of data circularity that affect the assessment of VEPs.

**Decision tree:** A model defining a series of rules that can be used to aid in classification by splitting the samples. Data entries are split until each ‘branch’ of the tree contains sufficiently homogenous data, belonging primarily to a single class.

**Deep mutational scanning (DMS):** A high-throughput wet-lab procedure that produces fitness scores for a high number of mutations of a protein. DMS is a type of multiplexed assay of variant effect (MAVE; see below). Protein fitness is linked to cellular growth rate or other quantifiable attributes and can be assessed by measuring the abundance of each variant through sequencing counts after growth.

**Dirichlet mixture model:** A statistical model that can be applied to estimate the frequency of unobserved amino acids at conserved positions during multiple sequence alignment.

**Fitness scores:** The endpoint of DMS is the generation of quantitative fitness scores for a high proportion of possible variants in a protein. These scores are often based on the change in sequencing counts of each variant during the course of an assay, which selects against variants that reduce fitness.

**Gold standard:** The currently most-reliable dataset for benchmarking that best reflects real-world observations.

**Holdout data:** The most common approach to reduce data circularity in predictor assessment is to withhold part of a dataset from predictor training. These unseen data are then used to assess the predictor performance.

**Indels:** Short insertions or deletions of <1000 nucleotides within the genome. Indels in protein-coding regions can result in frameshifts.

**K-fold cross-validation:** A method to assess the performance of a supervised predictor on its full training data without data circularity. The dataset is split into K equal-sized subsets, with one of them being used to test the predictor performance, while all others are used to train the predictor. This process is repeated by using each data subset to test the predictor performance, producing predictions for the complete training dataset.

**Meta predictors (ensemble predictors):** VEPs that use the outputs of other prediction algorithms to produce their own estimates of variant pathogenicity.

**Multiple sequence alignment (MSA):** A data structure produced by aligning amino acid positions of related proteins. This is done with the aid of a substitution matrix that defines the likelihood of certain amino acid substitutions. MSAs form the basis of all variant effect predictors.

**Multiplexed assay of variant effect (MAVE):** This term describes any large-scale experimental procedure that generates fitness scores for genetic variants. MAVEs that apply to protein-coding regions of the genome are deep mutational scans.

**Naïve metric:** A naïve approach to a problem is one that makes a broad, probably untrue, assumption to help simplify the problem. One example is the naïve Bayes classifier (Box 2), where all inputs are assumed to be fully independent from one another, although – in reality – this is rarely the case.

**NNK degenerate codons:** A nucleotide NNK codon, where N is any nucleotide and K is guanine or thymine. An NNK codon can encode any amino acid but only one STOP codon. The encoded amino acids are also depleted of those comprising many possible codons as compared to entirely random, i.e. NNN, codons.

**Position-specific independent counts (PSIC):** An algorithm that reduces the impact of redundant sequences in position-specific scoring matrices (see below). PSIC uses a statistical approach to weight-aligned sequences when generating an alignment profile.

**Position-specific scoring matrix (PSSM):** A matrix of weights that can be derived from a multiple sequence alignment based on the frequency of each amino acid or nucleotide at every aligned position. PSSM alignment profiles are a way to quantify conservation within an alignment and a useful way to represent alignment features in a machine learning algorithm.

**Pseudocounts:** Predictors that make direct use of conservation in a multiple sequence alignment, such as SIFT, are unable to directly determine the likelihood of a residue appearing at a certain aligned position if it is never present in the alignment. To overcome this issue, SIFT makes use of amino acid pseudocounts from a Dirichlet mixture model. These are theoretical frequencies of amino acids based on substitution scores in the BLOSUM62 substitution matrix.

**Receiver operating characteristic (ROC):** A probability curve that represents the ability of a classifier to distinguish between binary classes. The true positive rate is plotted against the false positive rate at varying thresholds. ROC curves can be summarised by the area under the curve (AUC), which is 1.0 for a perfect classifier, 0.5 for random guessing and 0.0 for a perfect inverted classifier.

**Variant effect predictors (VEPs):** Computational tools that use various different sources of information to predict the phenotypic outcome of genetic variants and help highlight variants that are most likely to have clinically relevant effects.

**Box 2. Machine learning techniques**

**Gradient-boosted trees** are similar to random forest algorithms in that they use an ensemble of decision trees to make predictions. Unlike random forests, trees constructed in gradient boosting are neither random nor independent but each new tree is constructed in the attempt to correct the errors of the previous one. The output of every new tree is added to the output of all previous trees and this process continues until a pre-determined maximum tree number is reached. Independently, each tree is a weak learner, performing barely better than random guessing but, together, they can solve complex problems.

**Neural Networks** are composed of ‘neurons’ that mimic the way biological neurons in the human brain communicate. Neurons in each layer of the network take inputs from the layer above, apply a function and pass the results to the next layer; a network with many stacked layers is a ‘deep’ neural network. Deep networks are capable of learning more-complex relationships between the input features but are harder to train. The network learns by comparing the output to the training labels and back-propagating the errors. Deep neural networks can learn to approximate extremely complex non-linear functions in order to separate classes based on the inputs.

**Naïve Bayes Classifiers** are simple, supervised algorithms that classify examples on the basis of a vector of features. As a naïve method, these classifiers assume that all their input features are independent in order to simplify the problem. They are based on the Bayes theorem that is used to determine the probability of a class label given prior knowledge. Naïve Bayes methods are often fast and effective but performance can degrade when too many features violate the assumption of independence.

**Random Forest (RF)** algorithms construct multiple decision trees by using a process called *bagging*. Each tree is trained to use a random selection of the available features and a random subset of the training data. Owing to the bagging process, each tree within the ensemble is different and, as independent models, they have a low correlation. Having multiple decision trees acts as a safeguard against overfitting and errors made by some of the trees. Classification is performed by majority vote.

**Support Vector Machines (SVMs)** are algorithms that separate two classes by constructing a *hyperplane* between them. This hyperplane is a line for 2D data, a plane for 3D data and so on. The hyperplane is placed so it has the largest possible distance to any instance of training data of both classes. More classes can be separated by the addition of further hyperplanes. Data that are not linearly separable can be classified by SVMs by using a non-linear kernel. Although SVMs have excellent classification performance, they function best with low-noise non-overlapping classes.

**Variational Autoencoders (VAEs)** are a class of unsupervised generative models. VAEs are composed of two neural networks. One of which is an *encoder* that takes the input and compresses it to a Gaussian distribution in latent space. The distribution is then sampled and a *decoder* neural network attempts to reconstruct the original input. VAEs are ‘generative’ because they can generate novel outputs based on the training data they have seen.

**Box 3. State-of-the-art predictors**

We make note of the following recently developed VEPs for their innovation in either methodology or choice of training datasets.

**Evolutionary Model of Variant Effect (EVE)** (Frazer et al., 2021) uses a methodology similar to that of its predecessor DeepSequence (Riesselman et al., 2018). Both are unsupervised methods utilising a variational autoencoder (Box 2) to learn the latent rules that underlie an MSA. No features other than those of MSA are provided for the method, and no pathogenic or benign training data are used. In principle, the latent rules learned by EVE are those that underlie the evolutionary process responsible for generating the MSA the predictor was trained with. Variant scores are determined by comparing the probability of these rules producing the mutant sequence and the probability of them producing the wild-type sequence.

**MutPred2** (Pejaver et al., 2020) uses a large number (1345) of features that can be categorised on the basis of sequence, substitution, position-specific scoring matrix (Box 1), conservation, homolog profiles or property changes. MutPred2 is a supervised, ensemble predictor composed of 30 separate neural networks that are trained using a matrix of features. A ‘bagging’ approach (Box 1), similar to how random forests are trained (Box 2), was used to expose each network to a random sample of training data. The predictions are the mean of the 30 neural network outputs. Training data have been derived from HGMD, SwissVar (discontinued) (Mottaz et al., 2010), dbSNP (https://www.ncbi.nlm.nih.gov/snp/) (Sherry et al., 1999) and interspecies alignments.

**VARITY** (Wu et al., 2021) makes use of the supervised gradient-boosted tree algorithm (Box 2) but innovates primarily in its use of unique training data. VARITY combines training examples from a large number of different sources, including functional assays. To compensate for potential low-quality data, training data are weighted based on specific metrics related to data quality, such as minor allele frequency within variant databases and internal quality metrics for functional assays.

## Variant effect predictors

### The importance of sequence conservation

Amino acid or nucleotide conservation (Box 1), calculated from alignment of related sequences, is an important feature for predicting variant pathogenicity. Random mutations are continually happening across the genomes of all species; those that are detrimental to organismal fitness are removed from the gene pool, while those that have no effect are much more likely to be propagated to the next generation. As species diverge, we observe that neutral substitutions build up in homologous proteins, such that sequence similarity reduces with evolutionary time (Kimura, 1983). Since pathogenic variants result in decreased fitness, these are far less likely to be present within an alignment of homologous proteins. Thus, we can assume that substitutions frequently observed within an alignment of sufficient depth are likely to be neutral in nature, whereas those absent or rarely observed are much more likely to be pathogenic.

Sequence conservation is fundamental to every VEP (Table 1). One of the simplest tools that can be built from an alignment is an amino acid substitution matrix, such as Blocks SubstitUtion Matrix (BLOSUM) (https://www.ncbi.nlm.nih.gov/Class/FieldGuide/BLOSUM62.txt) (Henikoff and Henikoff, 1992) or Point Accepted Mutations (PAM) (Dayhoff, 1972; Jones et al., 1992). These matrices are calculated directly from alignments and contain values representing the propensities for different amino acid substitutions among related sequences. Although they were originally intended as tools to aid the alignment of protein sequences, these simple approaches have been shown to have modest ability to predict pathogenic mutations (Rentzsch et al., 2019; Shauli et al., 2021). Under some conditions, substitution matrices can even outperform specialised VEPs (Chan et al., 2007; Livesey and Marsh, 2020).

**Table 1.**
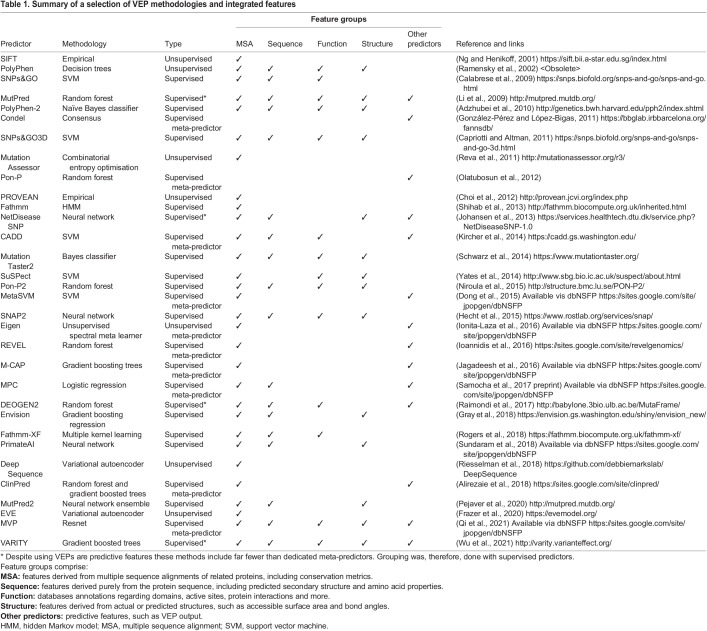
Summary of a selection of VEP methodologies and integrated features

Another method for measuring sequence conservation is by comparing the rate at which each amino acid (or underlying nucleotide) appears within a column of a multiple sequence alignment (MSA; Box 1) (Ng and Henikoff, 2001). Specialised nucleotide conservation metrics, such as Genomic Evolutionary Rate Profiling (GERP++, http://mendel.stanford.edu/sidowlab/downloads/gerp/index.html) (Davydov et al., 2010), PhyloP (http://compgen.cshl.edu/phast/) (Pollard et al., 2010) and Site-specific PHylogenetic analysis (SiPHy, https://portals.broadinstitute.org/genome_bio/siphy/index.html) (Garber et al., 2009) are often integrated into VEPs as measures of conservation. Such conservation metrics can also be used as standalone pseudo-predictors and have rivalled the performance of more-complex VEPs in some studies (Pejaver et al., 2020; Raimondi et al., 2017). Amino acid conservation is, therefore, a ‘proxy’ metric (Azevedo et al., 2017), but its utility for predicting pathogenicity is a testament to how effective the evolutionary process is at removing inefficient and pathogenic substitutions in nature.

### Early computational predictors

Substitution matrices set the groundwork for early VEPs, such as SIFT (Ng and Henikoff, 2001), which is still frequently used today for variant effect prediction (Table 1). However, with rapid advances in computing over the past decades, capability to execute complex algorithms and process large amounts of data has increased. Although SIFT has been outperformed in multiple recent benchmarking studies (Mahmood et al., 2017; Niroula and Vihinen, 2019; Thusberg et al., 2011), it has the advantages of rapidly returning results, being easy to interpret and simple to run.

SIFT functions by generating an MSA that is based on the protein-of-interest. Each column of the alignment is scanned to determine the frequency of substitutions and the probability that a specific substitution is tolerated at each position. Substitutions at residues with high levels of conservation are the most likely to be pathogenic. This process is similar to the derivation of the BLOSUM substitution matrices but uses an MSA generated specifically for the protein-of-interest; this makes the conservation position-specific to the protein, adding more context to the value returned. ‘Pseudocounts’ (Box 1), calculated from a Dirichlet mixture model (Box 1) (Sjölander et al., 1996), are added to the alignment to help compensate for amino acids not observed at certain positions. Prediction quality is dependent on the depth of the alignment and can vary significantly within and between proteins. SIFT is often taken as a point of comparison for modern predictors, which is a tribute to its popularity (Pejaver et al., 2020; Raimondi et al., 2017; Sundaram et al., 2018).

Polymorphism Phenotyping (PolyPhen) is another early VEP (Ramensky et al., 2002). Unlike SIFT, PolyPhen makes use of a large amount of non-sequence protein information. PolyPhen considers protein features that are derived from the amino acid substitution site, including secondary structure and database-derived key-site annotations, such as active site and binding sites. An MSA is used to generate position-specific independent count (PSIC; Box 1) profiles (Sunyaev et al., 1999). Finally, if the sequence can be mapped to a known 3D structure, additional features – such as site-proximity, accessible surface area and secondary structure – are also incorporated into the prediction. The original PolyPhen algorithm uses a decision tree (Box 1) to calculate a score for the mutation of interest. More recently, PolyPhen-2 was released (Adzhubei et al., 2010), a version that uses more features and replaces the empirically derived classification rules with a supervised naïve Bayes classifier (Box 2, Machine learning techniques).

**Fig. 1.**
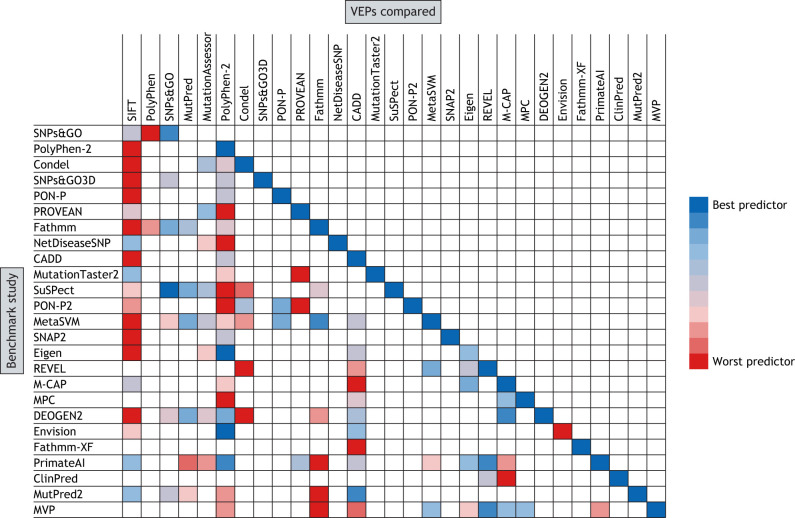
**Relative VEP performances in self-benchmarking analyses.** The VEPs at the left are those that published a benchmark in their method paper. The VEPs at the top were compared within these benchmarks. Owing to space constraints, we could not include all VEPs compared in each study. We took the reported performance metrics, such as ROC AUC, directly from each paper. These scores were then used to rank each predictor from best to worst performance in each benchmark. Where multiple performance metrics were available, we selected a single representative measurement – i.e. ROC AUC when possible – followed by balanced accuracy and then any other presented metric. In cases where multiple benchmarks were performed, we selected one that– if available – used data independently of VEP training or, if not, the most-prominent analysis within the paper. ROC AUC, receiver operating characteristic area under the curve.

Comparisons between SIFT and PolyPhen have found that, although each method has relatively high sensitivity, their specificity is low (Niroula and Vihinen, 2019). They are also much better at predicting loss-of-function than gain-of-function mutations (Flanagan et al., 2010). Both methods frequently appear inferior to many modern predictors in multiple benchmarking comparisons (Fig. 1) (Bendl et al., 2016; Ionita-Laza et al., 2016; Raimondi et al., 2016); however, they remain quick and straightforward to use, and their results are easy to interpret.

### Machine learning

The predictors summarised in Table 1 represent only a small number of available VEPs, with machine learning being the basis of most VEPs developed since PolyPhen in 2002. Machine learning aims to find patterns in features, such as conservation, secondary structure, amino acid properties and more, then use these to make predictions about pathogenicity. Compared to empirically calculated scores, using machine learning to automatically determine feature contributions allows for the inclusion of more sources of data in the calculation. Most often VEPs use a form of supervised machine learning.

Supervised machine learning methods learn by example, by training on labelled datasets. Databases collating pathogenic variants, such as ClinVar (https://www.ncbi.nlm.nih.gov/clinvar/) (Landrum et al., 2014) and the Human Gene Mutation Database (HGMD, http://www.hgmd.cf.ac.uk/) (Stenson et al., 2003), make ideal sources of pathogenic training examples, whereas gnomAD (https://gnomad.broadinstitute.org/) (Karczewski et al., 2020) provides a useful source of putatively benign variation observed in the human population. Several datasets exist, created specifically for the purposes of training and testing supervised VEPs, such as HumVar (http://genetics.bwh.harvard.edu/pph2/dokuwiki/downloads) (Capriotti et al., 2006) and Varibench (http://structure.bmc.lu.se/VariBench/) (Nair and Vihinen, 2013). These datasets also inevitably overlap by varying degrees (Mahmood et al., 2017). We highlight VARITY as an example of a state-of-the-art supervised predictor (Box 3, State-of-the-art predictors).

Many cutting-edge predictors use not only features calculated from protein sequences and structures but also the outputs of other VEPs. These meta-predictors, or ensemble predictors (Box 1), frequently combine features by using a supervised machine learning method, such as a random forest (Box 2) or a deep neural network (Box 2). Examples of these meta-predictors include ClinPred (Alirezaie et al., 2018), M-CAP (Jagadeesh et al., 2016), REVEL (Ioannidis et al., 2016) and MutPred2 that is highlighted in Box 3.

Unsupervised learning in VEPs has been slowly increasing in popularity, starting with the development of Eigen (Ionita-Laza et al., 2016). In unsupervised learning, the training examples are not labelled and the method makes its own decisions about how to make predictions. As the predictor does not see labelled examples during training, its predictions for specific variants are far less likely to be biased towards previous experience compared to supervised methods. Evolutionary Model of Variant Effect (EVE) is an example of an advanced unsupervised predictor (Box 3), but unsupervised learning shows even more promise as an avenue for future research.

## Benchmarking the performance of VEPs

### The problem of data circularity

Owing to the increasing volume of variants gathered in sequencing studies, with the majority being benign, identifying a single variant of concern in a noisy genetic background is extremely challenging, particularly if VEPs disagree as to the effect of the variant. As the amount of genetic data we generate keeps increasing, it is more important than ever to ensure we are using the correct tools for the job.

Benchmarking is most frequently performed by making predictions on sets of known pathogenic and benign variants. Relative performance is then assessed by several methods, most commonly by classification accuracy or area under the receiver operating characteristic curve (ROC AUC; Box 1). By far the most important aspect of benchmarking VEPs is the choice of variant dataset, which can substantially influence the outcome.

Grimm et al. (2015) described two types of data circularity in VEP benchmarking that can bias the assessment of predictor performance. Type 1 circularity primarily affects methods based on supervised machine learning. A method is susceptible to type 1 circularity if data used to train the model are re-used when assessing its performance. A model that is presented with data it has seen previously during training often performs better than it would if unseen data had been used. Whereas supervised machine learning methods are the most vulnerable to this form of bias, unsupervised methods are not immune. Such methods are often ‘tweaked’ based on their performance on a test dataset. This can lead to optimisation for that dataset and type 1 circularity if these variants are re-used while benchmarking. Type 2 circularity occurs because proteins with many variants in databases are often heavily skewed towards either pathogenic or benign outcomes. This results in deceptively good predictor performance if different variants from a single protein are used to train and test a predictor. However, in proteins that contain balanced numbers of pathogenic and benign variants, it can result in poor predictions.

Type 1 circularity is often addressed by careful curation of the variants used to benchmark predictors. However, this may limit the number of VEPs being compared if the training data of one fully overlaps the benchmarking dataset. Type 2 circularity is avoided by ensuring no variants in proteins used to train a predictor are used for benchmarking comparisons, ensuring that prior knowledge of variants in that protein is not used (Bromberg and Rost, 2007).

### Self-benchmarking

When reading papers describing new computational predictors, one will tend to encounter an interesting phenomenon: the authors will almost always find their own method to be better than any others. To illustrate this, we reviewed publications describing 25 new predictors (limited to those in Table 1), where their performance in predicting coding missense variants was assessed (Fig. 1). The self-benchmarking in these papers almost exclusively finds that the novel method is superior to its predecessors for general variant effect prediction. One exception is the unsupervised predictor Eigen, which underperformed PolyPhen-2 when the authors assessed the methods on missense variants – although Eigen did perform best when assessed on missense and nonsense variants combined (Ionita-Laza et al., 2016). In addition, Envision performed the worst in its internal benchmark against missense variants; however, it is primarily intended to predict mutagenesis data rather than pathogenicity, probably explaining its relative performance (Gray et al., 2018).

Although most methods perform consistently well within their own benchmarks, benchmarks from subsequent publications often disagree markedly regarding the relative performance of earlier predictors. For example, the authors of Fathmm (Shihab et al., 2013) find it to be the top-performing method among ten benchmarked predictors, including MutPred (Li et al., 2009). However, subsequent comparisons by other groups find Fathmm to underperform MutPred (Raimondi et al., 2017; Yates et al., 2014). There are also significant differences in performance when the same author performs multiple benchmarks. For example, DEOGEN (Raimondi et al., 2016) outperformed five other methods, including SIFT, MutationAssessor (Reva et al., 2011) and PolyPhen-2, when compared using the Humsavar 11 (https://www.uniprot.org/docs/humsavar) dataset for benchmarking (Raimondi et al., 2016). However, when using variants from an independent blind dataset, DEOGEN underperformed these three methods (Raimondi et al., 2017).

It is common for supervised machine learning-based VEPs to be benchmarked using their own training set, where predictions are generated by K-fold cross-validation (Box 1) to help prevent circularity (Adzhubei et al., 2010; Capriotti et al., 2013; Hecht et al., 2015; Pejaver et al., 2020). This may explain some of the performance discrepancies observed if it is the case that VEPs become optimised for the underlying biases of their training dataset. Furthermore, large variant datasets will inevitably have some underlying biases and structure, such as distinct proportions of variants from proteins with particular biological roles or disease mechanisms. Supervised predictors could use this information to over-perform in cross-validation or against holdout data (Box 1) (Capriotti and Altman, 2011; Carter et al., 2013; Jagadeesh et al., 2016) compared to tests using independent datasets. To overcome these issues, alternative benchmarking strategies include curated benchmarking (Box 1) datasets like Varibench (Feng, 2017; Niroula et al., 2015; Shihab et al., 2013; Yates et al., 2014) and making predictions on variants observed in relatively new studies that are unlikely to be present in any predictor training data (Dong et al., 2015; Raimondi et al., 2017; Sundaram et al., 2018).

One final issue with many benchmarking studies reported in VEP method papers is that certain well-known or innovative predictors are compared far more often than less-impactful VEPs that may still perform relatively well. SIFT and PolyPhen-2 have been compared in self-benchmarks of almost every VEP in the last 10 years (Fig. 1). SIFT, in particular, performs poorly in many of these comparisons but is still frequently used. In comparison, NetDiseaseSNP (Johansen et al., 2013) was only benchmarked in its own paper, so we have much less knowledge of how it compares to other predictors.

### Independent benchmarks

From the above, it is clear that we must refine benchmarking methods of VEPs to improve their reliability. For this reason, independent benchmarks of VEPs that reflect realistic use-cases are, potentially, far more useful comparisons than self-benchmarks. One of the earliest independent comparisons of VEPs (Thusberg et al., 2011) investigated nine predictors by using variants drawn from the Phencode database (http://phencode.bx.psu.edu/) (Giardine et al., 2007), locus-specific databases and dbSNP. Although the main comparison in the paper did not exclude any training data for the supervised methods, a subsequent, smaller scale comparison that only used data from the locus-specific databases found that all methods performed worse on the limited dataset. No single predictor was superior by all outcome metrics; however, SNPs&GO (Capriotti et al., 2013) produced the highest accuracy for both the main study and on the limited dataset.

Most other early independent benchmarking studies focused on a small number of variants within a single protein or a group of related proteins. For example, VEPs were compared by using independent benchmarking in studies of 51 variants in the bilirubin uridine diphosphate glucuronsyltransferase gene (*UGT1A1*) (Galehdari et al., 2013), 74 variants in DNA mismatch repair genes (Thompson et al., 2013) and 122 RASopathy variants (Walters-Sen et al., 2015). In the *UGT1A1* study, SIFT performed best in terms of classification accuracy, despite being the oldest method. The DNA mismatch study acknowledged the issue with potential circularity, particularly in MutPred and PolyPhen-2, which were trained with variants in the target proteins. Re-training these predictors without variants in the mismatch repair genes resulted in degraded performance, particularly for MutPred. Unlike the other predictors in the study, SIFT allows the user to provide their own MSA rather than relying on the tool's native alignment. Interestingly, this group found that supplying hand-curated alignments featuring full-length homologues and sufficient variation at all positions to SIFT markedly improved performance over its native alignments. This demonstrates that the quality of the alignment used by VEPs has a significant impact on the predictions generated. The RASopathy study investigated 15 predictors, finding that the majority of programs performed below their published level. This study did not exclude any variants used to train the VEPs; therefore, even the predictors most strongly influenced by type 1 data circularity still performed poorly on this dataset.

The study by Grimm and colleagues (Grimm et al., 2015) highlighting the issue with data circularity, also contained benchmarks that adhere to the principles of minimising circularity by carefully selecting variants from Varibench, predictSNP (https://loschmidt.chemi.muni.cz/predictsnp/) (Bendl et al., 2014) and SwissVar, which were not used to train any of the assessed VEPs. Grimm et al. found that, when some of the training data for predictors are present within the benchmarking set, most supervised methods performed at their best. Performance degrades when all training data are excluded from the test. When the issue of type 1 circularity is not a factor, the empirically derived SIFT is comparable to some supervised machine learning methods, such as CONDEL (González-Pérez and López-Bigas, 2011). The approach used in this paper was highly effective at eliminating type 1 circularity by curating variant databases. However, as more predictors are trained, such datasets will need to be continuously refined and predictor training data always made public to allow such comparisons in the future. Private datasets, such as HGMD, make these measures difficult to implement.

Modern benchmarking studies often take a similar approach to Grimm et al. (2015) in curating the assessment data to remove any VEP training variants and limit the number of VEPs being compared. A recent study comparing five predictors on clinical variants (Gunning et al., 2021) used data from ClinVar, HGMD, Online Mendelian Inheritance in Man (OMIM, https://www.ncbi.nlm.nih.gov/omim) and gnomAD, in addition to clinical and population studies to benchmark the VEPs. Although no training data were present in the variants used for the assessment, all tools – including SIFT – performed better on the dataset derived from existing database entries than on the dataset from newer clinical and population studies. Since type 1 circularity was not an issue, it is clear that the predictors are still optimised to better predict variants in the open dataset. One implication is that pathogenicity thresholds for VEPs might not be consistent between datasets, necessitating calibration studies. Such studies would involve testing VEPs against multiple datasets of variants or even individual proteins to determine the optimal prediction thresholds of each method in different contexts. Another recent study focussed on the often-overlooked ability of VEPs to predict benign variants (Niroula and Vihinen, 2019). To compare ten different predictors, this study used common variants from the former ExAC database that is now available at gnomAD (https://gnomad.broadinstitute.org/) (Karczewski et al., 2017). Training data from the supervised VEPs were filtered out of the ExAC data; however, the training data of four predictors – MetaLR, MetaSVM (Dong et al., 2015), M-CAP and REVEL – fully overlapped with the ExAC data, resulting in their exclusion from the study. This highlights that such studies are limited in the number of predictors they can assess while still accounting for data circularity.

One solution adopted by several groups to resolve the issue of data circularity is to use a gold standard (Box 1) for assessment, which is fully independent of any existing training data. This can be achieved by using datasets derived from experimental assays of variant effect. The use of independently generated functional data reduces the need to rely on databases that overlap VEP training data. Mutagenesis experiments also have the potential to assess the function of many entirely novel variants. Until recently, however, such comparisons were only possible on a small scale.

## Deep Mutational Scanning

### Experimental procedure

DMS is a relatively new fusion of large-scale mutagenesis and high-throughput sequencing that provides quantitative measurements of variant fitness, potentially assessing all possible variants in a protein (Fowler and Fields, 2014). This is a vast improvement over previous mutagenesis and directed evolution studies that focus on only a small subset of possible variants. DMS technology has rapidly improved, culminating in several studies that accurately recapitulate the effects of clinically validated variants (Findlay et al., 2018; Mighell et al., 2020).

**Fig. 2.**
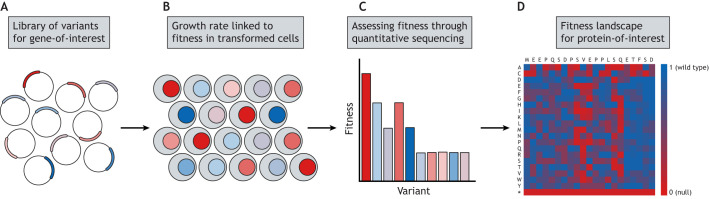
**Summary of a typical DMS experiment.** (A) A library of variants, often representing every possible amino acid substitution in a protein, is generated and cloned into expression vectors. (B) The vectors are then introduced to mammalian or yeast cells where the function of the mutant protein is linked to the cell growth rate or some other measurable attribute. (C) Variant fitness is measured at different time points by quantitative sequencing, and compared to positive and negative controls to calculate relative fitness values. (D) A fitness map of all possible variants in the protein can be constructed from the relative fitness data.

All DMS studies begin with the generation of a library of mutant genes, usually accounting for all possible amino acid variants in the protein of interest (Fig. 2A). One such technique is POPCode (Weile et al., 2017), allowing replacement of each codon in the gene by using mutagenic primers with NNK degenerate codons (Box 1). Alternatives include a variety of techniques, from direct synthesis of the variant library (Jones et al., 2020) to random mutagenesis by error-prone PCR (Choudhury et al., 2020).

To assess the fitness of each variant in the library, protein function has to impact some measurable attribute of the cells expressing the mutant proteins. The exact mechanism varies greatly between experiments but, in the simplest case, the fitness of variants can be linked to cell growth rate (Fig. 2B) (Brenan et al., 2016; Giacomelli et al., 2018; Weile et al., 2017). Expressing variants that are unable to perform their function as effectively as the wild type reduces growth rates. As a final step, the growth rate effects of each variant can be determined by quantitative sequencing at different time points throughout the assay (Fig. 2C). Each variant is assigned a fitness score relative to the wild type and a null control. DMS experiments ultimately produce a heatmap of measured variant fitness over the protein or domain assessed at each position, indicating the areas that are least tolerant of substitutions (Fig. 2D).

The goal of DMS is to understand how different amino acid substitutions affect the ability of a protein to function. Where function of the protein is vital for cell growth or survival, it is simple to relate the resulting functional scores to disease risk. In proteins with a less-direct link to disease, protein function might need to be linked to cell growth rate through an artificial mechanism, such as placing an essential gene under the control of a yeast two-hybrid construct to assess protein–protein interaction fitness (Bandaru et al., 2017; Starita et al., 2015). In these cases, there is no guarantee that the measured fitness metric will be correlated with human disease risk.

### Using DMS to predict clinical outcomes

Potentially the most-exciting use of DMS is in directly assessing the clinical impact of novel variants, a feature it shares with VEPs. Unlike supervised VEPs, however, DMS fitness values are derived independently of previous data and, thus, are not subject to the circularity issues that VEPs are vulnerable to. DMS has shown promise in separating ClinVar pathogenic variants from putatively benign gnomAD variants, outperforming VEPs for several proteins (Livesey and Marsh, 2020).

Numerous germline variants in the tumour suppressor *BRCA1* have been found to pre-dispose women to developing breast cancer. Despite many sequencing studies, there still remains a large number of *BRCA1* VUSs and many novel variants to be observed. A recent DMS study quantified the effects of nearly 4000 *BRCA1* variants (Findlay et al., 2018). The group used a human HAP1 cell line and its growth rate as a fitness measure, owing to the essentiality of *BRCA1* for growth in this cell line (Blomen et al., 2015). The DMS results showed extremely high concordance with annotated ClinVar variants. Furthermore, Findlay et al. provided evidence that, for some variants where ClinVar and the DMS data diverge, DMS may provide a more-accurate functional assessment.

*PTEN* is another gene with numerous cancer-related germline variants and links to neurodevelopmental disorders. It is a tumour suppressor gene with a large number of benign variations, making it an excellent target for DMS studies. One study assessing *PTEN* variant fitness used a yeast system, in which *PTEN* activity rescues cell growth (Mighell et al., 2018). The authors found that the DMS data separated pathogenic ClinVar from putatively benign gnomAD variation with high levels of accuracy and sensitivity. This atlas of experimentally assessed variants may be useful for identifying potentially pathogenic mutations, and for distinguishing variants resulting in cancer from those causing neurodevelopmental disorders (Mighell et al., 2020).

Overall, these examples show that well-constructed DMS experiments can accurately identify known pathogenic variants, and have potential to annotate novel variants and VUS in disease-related genes. Such experiments – rather than clinical observations – may even have the potential to become a new ‘gold standard’ for variant outcome assessment.

### Using DMS to assess the performance of VEPs

The Critical Assessment of Genome Interpretation (CAGI) is an ongoing experiment to assess the state of VEPs and related software (Andreoletti et al., 2019). In CAGI, software is tested in a series of variant interpretation challenges, spanning single nucleotide variants (SNVs), indels (Box 1), different molecular phenotypes, splicing effects, regulatory elements and more. CAGI assesses progress in the field by using data held back from publication, so that methods cannot be trained using these data. The 5th edition of experiments, i.e. CAGI5 challenge, included challenges derived from DMS-type experiments that included a yeast complementation assay with human calmodulin (Zhang et al., 2019), and a thermodynamic stability assay of *PTEN* and thiopurine S-methyltransferase (Matreyek et al., 2018).

Beyond CAGI, several independent groups have also applied data from DMS-style functional assays to benchmark VEPs. In an attempt to benchmark 46 VEPs, our lab previously used functional data from 31 DMS datasets from human, yeast, bacterial and viral sources (Livesey and Marsh, 2020). We calculated a relative rank score for each predictor based on the Spearman's correlation between the continuous scores output by the VEPs and the DMS datasets. Overall, we found that the unsupervised method DeepSequence showed the best performance for human and bacterial proteins. SNAP2 (Hecht et al., 2015), DEOGEN2 (Raimondi et al., 2017), SuSPect (Yates et al., 2014) and REVEL also displayed relatively high performance, as well as ease of querying. Although variants used to train predictors were not removed from the benchmarking data, they made up only a tiny fraction of the overall DMS dataset.

Another study used three datasets composed of published *BRCA1* DMS data, *TP53* DMS data and variants in UniProt that originated from human mutagenesis experiments (UniFun). These three datasets overlap only very slightly with common training datasets (Mahmood et al., 2017). Compared to data commonly used for benchmarking, the BRCA1 and UniFun variants were poorly predicted, whereas accuracy regarding *TP53* data was relatively high for many methods. Mahmood and colleagues concluded that the difference in performance between traditional benchmarking datasets and functionally derived data is probably due to data circularity, providing an advantage on the former. An interesting consequence is that the empirical SIFT method produced the best performance on the UniFun data, outclassing multiple supervised machine learning methods.

Data from 22 DMS experiments were used to evaluate four VEPs together with conservation metrics (Reeb et al., 2020). Overall, Envision most accurately predicted variant deleteriousness determined by DMS. It was, however, unclear whether this was owing to genuine benefits of the VEP or bias due to Envision being trained directly using DMS data. The output of all VEPs tested correlated slightly with the DMS fitness values; however, all methods performed better on deleterious SNVs than on beneficial (gain-of-function) mutations. A naïve metric (Box 1) using PSI-BLAST also performed surprisingly well, outperforming some VEPs for classification.

DMS is a source of experimentally validated variant effect scores that are fully independent from existing classifications in databases most often used to train VEPs. This independence, together with the presence of large numbers of novel variants, allows for benchmarking of more predictors than traditional studies with less risk of data circularity. We can expect the popularity of using such datasets as a benchmark to increase, as DMS datasets become available for even more proteins.

## Conclusions

VEPs and DMS studies can both be used to identify potentially pathogenic variants. With DMS technology constantly improving and sequencing becoming cheaper, the question arises whether we will need VEPs in the future, if DMS can provide us with direct measurements of variant effect. The precise definition of ‘fitness’ in DMS is very important as a protein's fitness can often be defined in multiple ways. The challenge is to measure fitness in a way that correlates best with clinical outcomes. It is not always obvious how to achieve this for every protein. Although DMS results reflect the clinical outcome for many variants, for others the correlation can be poor (Livesey and Marsh, 2020). In the latter, the assessed fitness metric probably did not adequately reflect the mechanisms behind the disease caused by mutations of those proteins. However, DMS is also possible for proteins without disease association if fitness can still be assessed in some way. VEP benchmarking performance on such data is likely to dependent on whether the predictor takes the protein role and context into account for scoring.

Owing to their fast generation time and genetic tractability, many DMS studies are carried out in yeast cells. There is some concern that, because of evolutionary differences, fitness scores from yeast cells might not accurately reflect human genetic disease outcomes. Despite these differences between humans and yeast, it has been demonstrated that functional complementation assays performed in yeast systems still manage to accurately predict human disease (Sun et al., 2016).

However, DMS is resource-intensive and expensive, which can limit its use as a benchmark. A considerable level of expertise is also required to devise a suitable fitness assay and troubleshoot unforeseen issues. It is not currently feasible to perform DMS for every protein and, even if we could, there is no guarantee that the measured fitness metric would be applicable to human disease prediction. Until we have the knowledge and resources to construct assays to take into account all possible definitions of protein fitness, there will still be a requirement for VEPs in the future.

New generations of VEPs are constantly expanding their training data to broaden their experience and, hopefully, produce more accurate results. Recently, some VEPs that include DMS data as part of their training sets have been published. Benchmarking such predictors using DMS data carries the same caveats as benchmarking other supervised predictors with commonly used variant databases. For these VEPs, data circularity becomes an issue once again and, although dataset curation may help prevent circulatory, optimisation for DMS datasets may give the predictor an unfair advantage regardless. The two predictors we are aware of that make use of DMS data in their training sets are Envision and VARITY. Envision has been found to accurately predict effect magnitude in DMS datasets (Reeb et al., 2020), although our group found that Envision produced an average performance by using a different set of DMS data (Livesey and Marsh, 2020). The ability of VARITY to predict DMS data has yet to be assessed, although a study using gene-trait combinations found it to have excellent predictive performance (Kuang et al., 2021).

The question, therefore, remains whether functional assays can solve the issue of data circularity if variants used to train the VEPs are not excluded from the benchmarking dataset. The key is in the complete independence of DMS-derived datasets from variant interpretations based on clinical observation. Although there is usually a strong correlation, the measured variant effects in DMS are not necessarily identical to those in variant databases. DMS results are also often non-binary continuous values, which allows for differentiation between ‘extremely damaging’ and ‘slightly damaging’ variants, a distinction not found in traditional variant databases. Furthermore, the correlation between VEP predictions and measured intra-protein variant effects is likely to help in identifying those predictors susceptible to type-2 circularity. As previously outlined, type 2 circularity is caused by VEPs that associate particular proteins with a pathogenic or benign outcome and then applying that knowledge to new variants in the same protein. This effect provides the VEP with an advantage for binary classification of variants. In order to perform well against DMS fitness scores, a VEP has to have a strong correlation with the continuous values – which cannot be obtained solely by using knowledge of protein–disease associations. Overall DMS data stand as a potential solution to data circularity, particularly as more datasets emerge. However, this may soon become complicated by a new generation of VEPs trained with DMS data.

In this Review, we have described the recent progress made on computational predictors of variant effect and the issues with benchmarking these programs. DMS stands as not only a useful independent source of benchmarking data to limit circularity from performance estimates but also a, potentially, useful resource for direct variant classification. Outside of direct variant-effect prediction, DMS can also be applied to protein structure prediction (Adkar et al., 2012), residue contact prediction (Sahoo et al., 2015) and protein engineering (Spencer and Zhang, 2017).

We hope that, in future works, variant effect datasets from DMS-type studies will be more widely used to assess the performance of VEPs. This trend should naturally arise as further DMS experiments are carried out on human proteins and as the evolution of DMS methodology continues. With such datasets being used, we also expect to see an increase in the scope of independent benchmarking studies to include additional predictors. Such advances are likely to assist the identification of the best predictor methodologies and aid the production of more-accurate VEPs.
